# A novel application of motion analysis for detecting stress responses in embryos at different stages of development

**DOI:** 10.1186/1471-2105-14-37

**Published:** 2013-02-01

**Authors:** Oliver Tills, Tabitha Bitterli, Phil Culverhouse, John I Spicer, Simon Rundle

**Affiliations:** 1 Marine Biology and Ecology Research Centre, School of Marine Science and Engineering, Plymouth University, Plymouth, Devon PL4 8AA, UK; 2 Centre for Robotics and Neural Systems, School of Computing and Mathematics, Plymouth University, Plymouth, Devon, PL4 8AA, UK

**Keywords:** Motion analysis, Bioimaging, Toxicology, Developmental stages

## Abstract

**Background:**

Motion analysis is one of the tools available to biologists to extract biologically relevant information from image datasets and has been applied to a diverse range of organisms. The application of motion analysis during early development presents a challenge, as embryos often exhibit complex, subtle and diverse movement patterns. A method of motion analysis able to holistically quantify complex embryonic movements could be a powerful tool for fields such as toxicology and developmental biology to investigate whole organism stress responses. Here we assessed whether motion analysis could be used to distinguish the effects of stressors on three early developmental stages of each of three species: (i) the zebrafish *Danio rerio* (stages 19 h, 21.5 h and 33 h exposed to 1.5% ethanol and a salinity of 5); (ii) the African clawed toad *Xenopus laevis* (stages 24, 32 and 34 exposed to a salinity of 20); and iii) the pond snail *Radix balthica* (stages E3, E4, E6, E9 and E11 exposed to salinities of 5, 10 and 15). Image sequences were analysed using Sparse Optic Flow and the resultant frame-to-frame motion parameters were analysed using Discrete Fourier Transform to quantify the distribution of energy at different frequencies. This spectral frequency dataset was then used to construct a Bray-Curtis similarity matrix and differences in movement patterns between embryos in this matrix were tested for using ANOSIM.

**Results:**

Spectral frequency analysis of these motion parameters was able to distinguish stage-specific effects of environmental stressors in most cases, including *Xenopus laevis* at stages 24, 32 and 34 exposed to a salinity of 20, *Danio rerio* at 33 hpf exposed to 1.5% ethanol, and *Radix balthica* at stages E4, E9 and E11 exposed to salinities of 5, 10 and 15. This technique was better able to distinguish embryos exposed to stressors than analysis of manual quantification of movement and within species distinguished most of the developmental stages studied in the control treatments.

**Conclusion:**

This innovative use of motion analysis incorporates data quantifying embryonic movements at a range of frequencies and so provides an holistic analysis of an embryo’s movement patterns. This technique has potential applications for quantifying embryonic responses to environmental stressors such as exposure to pharmaceuticals or pollutants, and also as an automated tool for developmental staging of embryos.

## Background

The development of imaging technology over the past two decades has enabled significant advances in biology and resulted in an exponential increase in the size and complexity of the data sets generated [1,2]. However, this ability to acquire data at a rapid rate has also led to major data management challenges [3]. Consequently the ability to manage and analyse image sequences, particularly those recorded at a high frame rate, is now recognised as the major bottleneck in laboratories using high throughput imaging systems [4,5]. This bottleneck is being addressed by the development of bioinformatics, an emerging field tasked with developing techniques to extract biological data from image datasets allowing the subsequent deletion of memory intensive images [6].

Among the many methods of automated image analysis available to biologists is motion analysis, a technique that has been used for quantifying the behavior of mammals [7], fish [8], sperm [9], larvae [10], algal spores [11,12] and bacteria [13]. These applications measure parameters including distance travelled, speed and angular velocity and they therefore rely on an observable forward motion in the sample studied. The application of these motion analysis systems to quantify embryonic movement effectively is limited, as the movement patterns embryos exhibit are often complex, subtle and diverse (e.g. muscle flexing, spinning, heart beat, blood flow and tail flicking), which creates challenges in quantifying embryonic movements in a holistic way. Here we demonstrate a technique for quantifying motion patterns in embryos that can distinguish the effects of environmental stressors on embryos at different developmental stages.

**Figure 1:**
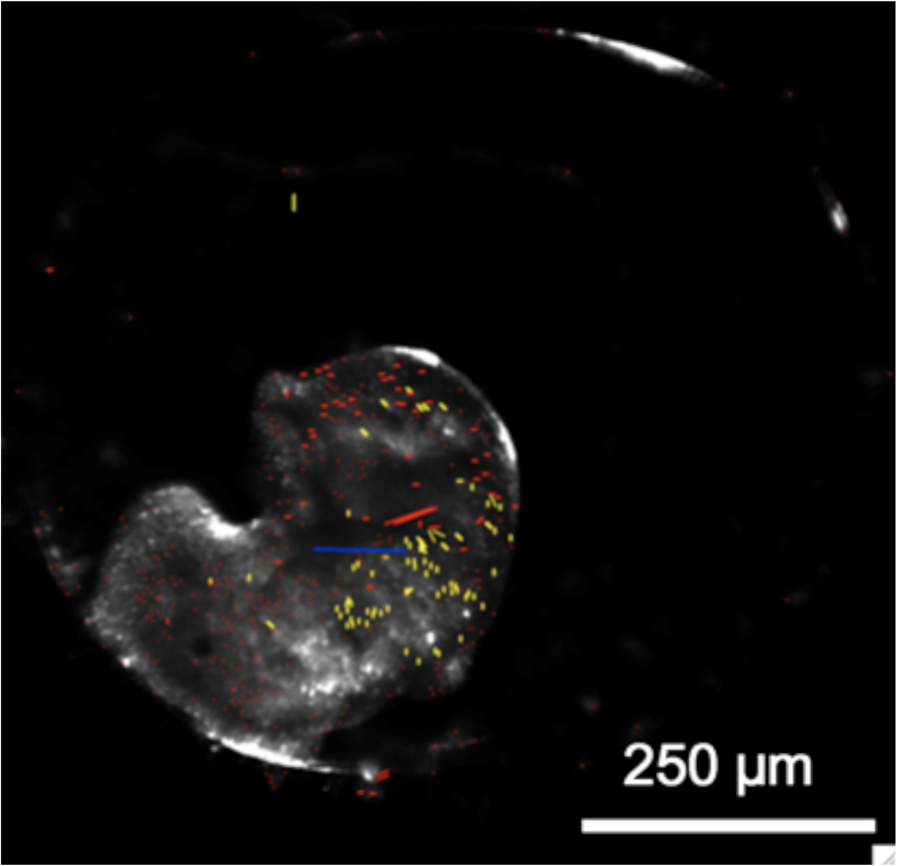
**Optic flow of an E6 stage *Radix balthica *embryo showing angular rotations (red and yellow) and centre of mass (blue line).**

We applied sparse optic flow [14], a machine vision motion analysis technique (Figure 1), to image sequences of two widely used vertebrate model species at three early developmental stages: (i) *Danio rerio* embryos at stages 19 h, 21.5 h and 33 h [15], and (ii) *Xenopus laevis* at stages 24, 32 and 34 [16]; and (iii) to embryos of an invertebrate, the freshwater pond snail *Radix balthica*, at stages E3, E4, E6, E9 and E11 [17]. At each developmental stage image sequences were recorded under control conditions and in embryos exposed to abiotic stressors, for which developmental effects have previously been observed [18-26]. *Danio rerio* embryos were exposed to 1.5% ethanol [18 – 21], and a salinity of 5 [22-24], *Xenopus laevis* were exposed to a salinity of 20 [25] and *Radix balthica* embryos were exposed to salinities of 5, 10 and 15 [26].

**Figure 2:**
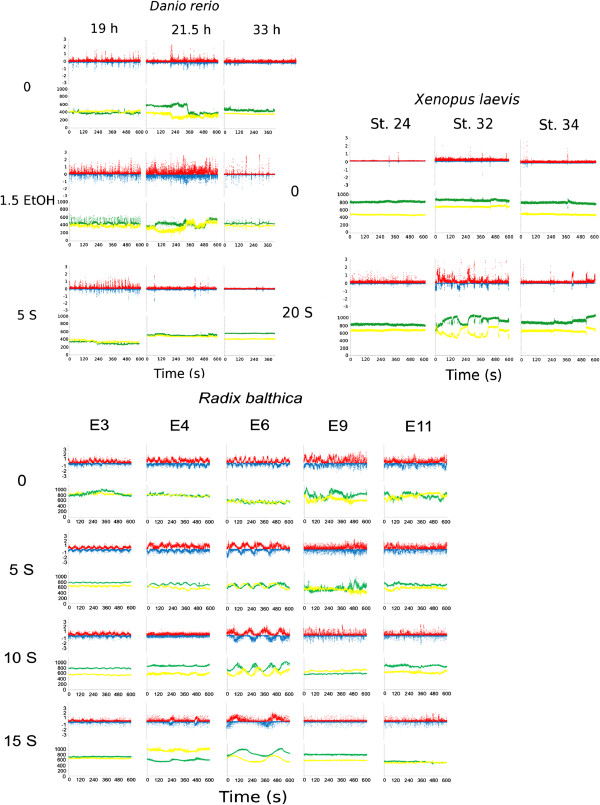
**Frame by frame optic flow parameters of control and treatment embryos for each of the developmental stages of *Danio rerio*, *Xenopus laevis *and *Radix balthica*. **Red – positive angle movements, blue – negative angle movements, green – X coordinate of centre of mass, yellow – Y coordinate of centre of mass. A time period (*Danio rerio *– 10 min, *Xenopus laevis *– 10 min, *Radix balthica *– 5 min) of a single embryo is shown, exhibiting typical movement patterns for each treatment response per developmental stage.

**Figure 3:**
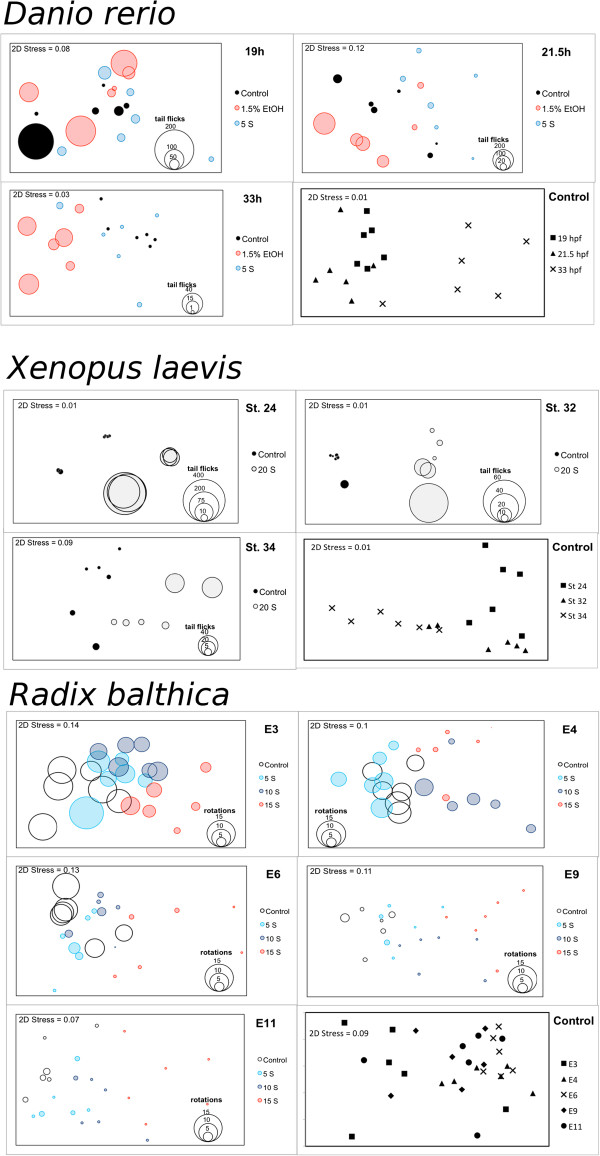
**Multidimensional scaling plots in two dimensions using Bray-Curtis similarity matrices. **MDS plots using Bray-Curtis similarity matrices performed on logarithmically transformed spectral frequency data produced using Discrete Fourier Transform analysis of (i) negative angle (ii) positive angle (iii) centre of mass – rho and (iv) centre of mass – theta, frame-to-frame parameters for (**a**) *Danio rerio*, (**b**) *Xenopus laevis *and (**c**) *Radix balthica *under different environmental conditions and at different developmental stages. Bubble size represents the amount of tail flicks of *Danio rerio *and *Xenopus laevis*, the number of rotations of *Radix balthica* at stages E3, E4 and E6, and the number of complete gliding along the circumference of the egg capsule on stages E9 and E11.

As the embryos are shape-deformable it is sensible to encode optic flow as a summation of directed rotations. Optic flow was therefore calculated as the mean angles of positive (clockwise) and negative (anti-clockwise) rotation for each frame and, from these data, the centre of mass was calculated as X and Y polar coordinates (Figure 1). The patterns in these frame-by-frame movement parameters (Figure 2) were analysed using Discrete Fourier analysis, run using the R language for statistical computing [27], to quantify the distribution of energy of each of these parameters, within 18 to 30 frequency bins ranging between 600 – 0.1 s. A Bray-Curtis similarity matrix based on 72 variables (18 frequency bins for each of the parameters - mean angles of positive and negative rotation and the X and Y coordinates of the calculated centre of mass) was visualised using Multidimensional Scaling (Figure 3) and differences in movement patterns between developmental stages and treatment groups were tested statistically using ANOSIM [28] (Tables 1 and 2). This analysis revealed that, in all three species, for most of the developmental stages examined, individuals exposed to an environmental stressor were distinguishable from control organisms and also that many of the developmental stages in control treatments were distinguishable from each other. This technique could be applied to any species whose movement patterns can be visualized and could have applications within the fields of biotechnology, toxicology and developmental biology where the extraction of a holistic measure of embryonic movement patterns would be desirable.

**Table 1:** **Comparison of analysis of treatment groups by both the automated motion analysis technique** and observer quantification

***a) Danio rerio***							
**Developmental stage**	**Treatment**	**5 salinity**		**Control**			
		**Automated**	**Manual**	**Automated**	**Manual**		
19 hpf	**Control**	ns	ns				
	**1.5% EtOH**	ns	ns	ns	ns		
21.5 hpf	**Control**	ns	ns				
	**1.5% EtOH**	*	*	ns	ns		
33 hpf	**Control**	ns	ns				
	**1.5% EtOH**	*	ns	***	**		
***b) Xenopus laevis***							
**Developmental stage**	**Treatment**	**20 salinity**					
		**Automated**	**Manual**				
St. 24	**Control**	***	*				
St. 32	**Control**	***	ns				
St. 34	**Control**	***	ns				
***c) Radix balthica***							
**Dev. stage**	**Treatment**	**Control**		**5 salinity**		**10 salinity**	
		**Automated**	**Manual**	**Automated**	**Manual**	**Automated**	**Manual**
E3	**5 salinity**	*	ns				
	**10 salinity**	**	***	ns	ns		
	**15 salinity**	**	***	**	*	**	*
E4	**5 salinity**	*	*				
	**10 salinity**	**	***	**	ns		
	**15 salinity**	**	***	**	***	*	*
E6	**5 salinity**	**	***				
	**10 salinity**	**	***	ns	ns		
	**15 salinity**	**	***	**	ns	**	ns
E9	**5 salinity**	ns	ns				
	**10 salinity**	**	*	*	ns		
	**15 salinity**	**	*	**	ns	*	ns
E11	**5 salinity**	*	ns				
	**10 salinity**	**	*	*	ns		
	**15 salinity**	**	***	**	*	**	*

ANOSIM of Bray-Curtis similarity matrices calculated from the results of Discrete Fourier Transform on frame-to-frame motion parameters. Manual quantification of movement patterns: number of tail flicks for *Danio rerio *(19 hpf - F_17_ = 1.23, P = 0.321; 21.5 hpf - F_17 _= 4.70, P = 0.026; 33 hpf - F_17_ = 11.83, P = 0.001) and *Xenopus laevis *(St. 24 – F_11_ = 8.98, P = 0.013; St. 32 – F_11 _= 3.38, P = 0.096; St. 34 – F_11 _= 3.16, P = 0.106) and number of embryo rotations for *Radix balthica *(E3 – F_23 _= 9.36, P = ≤ 0.001; E4 – F_23 _= 26.33, P = ≤ 0.001; E6 – F_23 _= 58.25, P = ≤ 0.001; E9 – F_23 _= 8.40, P = 0.001; E11 – F_23_ = 7.88, P = 0.001); ANOVA between treatment groups to tests for differences in the manual quantification of embryonic movement for (a) *Danio rerio*, (b) *Xenopus laevis *and (c) *Radix balthica*. *** - p ≤ 0.001, ** - p ≤ 0.01, * - p ≤ 0.05, ns – not significant.

**Table 2:** Significance levels, calculated using ANOSIM, of pairwise differences in developmental stages

**(a) *Danio rerio***				
19 h	**			
33 h	**		**	
	21.5 h		19 h	
**(b) *Xenopus laevis***				
St. 24	ns			
St. 34	**		**	
	St. 32		St. 24	
**(c) *Radix balthica***				
E4	**			
E6	**	**		
E9	ns	*	ns	
E11	*	**	**	*
	E3	E4	E6	E9

Using Bray-Curtis similarity matrices calculated from the Discrete Fourier Transform of frame-to-frame motion parameters for (a) *Danio rerio *(b) *Xenopus laevis *and (c) Radix balthica. *** - p ≤ 0.001, ** - p ≤ 0.01, * - p ≤0.05, ns - not significant.

## Results

Time series of the output parameters produced by optic flow (Figure 2) revealed patterns that accurately reflected the organism’s movement seen during manual observation of the same image sequence, e.g. tail flicks in zebrafish embryos resulted in peaks in the motion parameters recorded by the optic flow motion analysis. Subsequent analysis of the frame-to-frame motion analysis parameters was successful in distinguishing individuals exposed to environmental stress from those under control conditions within many of the developmental stages studied (Table 1), and to distinguish, within species, individuals under control conditions at different stages of development (Table 2).

### *Danio rerio*

*Danio rerio* embryos at 33 hpf exposed to 1.5% ethanol were statistically distinguishable from those under control conditions (Table 1). All developmental stages of *Danio rerio* used here (19, 21.5, 33 hpf) exhibited increased tail flick frequency in response to exposure to 1.5% ethanol and this was visible in the optic flow motion parameter plots as increased peak frequencies (Figure 2). Embryos exposed to 1.5% ethanol also exhibited more vigorous tail flicking and this caused greater levels of movement within the egg than seen in embryos under control conditions, recorded by optic flow as changes to the calculated X and Y coordinates of the centre of mass. However, embryonic movement responses at stages 19 and 21.5 hpf were highly variable. Two embryos from both 19 and 21.5 hpf showed movement patterns typical of control embryos and one embryo at 19.5 hpf exposed to ethanol performed more tail flicking (182 tail flicks in 10 min) than all other embryos exposed to ethanol combined. This inter-individual variation perhaps goes someway to explaining why stress responses in these developmental stages were not distinguishable. Exposure to a salinity of 5 produced no discernible difference in movement responses compared with control embryos.

*Danio rerio* at 19, 21.5 and 33 h post fertilization (hpf) under control conditions were all distinguishable from each other via analysis of frame-to-frame parameters. *Danio rerio* at 19 h post fertilisation (hpf) performed a regular flicking at the end of the tail. This movement resulted in optic flow producing small peaks in the magnitude of clockwise and anti-clockwise movements and in the calculated X and Y coordinates of the embryo’s centre of mass. Embryos at 21.5 hpf performed a less regular tail flicking involving a greater length of the embryo’s tail and this was visible in the motion analysis data as peaks in clockwise and anticlockwise movements and also in the X and Y image coordinates of the embryo, with greater amplitude than those seen in 19 hpf embryos. The more vigorous embryonic movements caused occasional shifts in the orientation of the embryo within the egg and this resulted in periodic shifts in the X and Y image coordinates of the embryo. *Danio rerio* at 33 hpf exhibited the longest periods with no gross movement, but when embryos at this stage did perform tail flicks these were more vigorous than at 19 or 21.5 hpf and so this produced larger peaks in the clockwise and anticlockwise movements recorded. There was little change in the X and Y coordinates of the centre of mass in *Danio rerio* at 33 hpf due to the embryo having little space to move within the egg capsule.

### *Xenopus laevis*

*Xenopus laevis* exposed to a salinity of 20 in the three developmental stages used here were statistically distinguishable from control individuals (Table 1; Figure 3). *Xenopus laevis* at stage 24 exhibited an increase in tail flicking frequency when exposed to a salinity of 20, although there was significant inter-individual variation in this response ranging from 48 to 400 tail flicks, during the ten minute recording period. Despite this variation there was a clear distinction in the Multidimensional Scaling plot between control and salinity exposed embryos (Figure 3). *Xenopus laevis* at both stages 32 and 34 exhibited more frequent tail flicking when exposed to the salinity treatment. Tail flicking in stage 32 embryos exposed to salinity caused movement of the egg capsule within the well and this was observed in the motion analysis as changes to the calculated X and Y centre of mass parameters.

*Xenopus laevis* under control conditions at stage 34 was distinguishable from both stages 32 and 24, however stage 24 was not distinguishable from 32. *Xenopus laevis* at stage 24 performed only a very occasional tail flicking and this was a slow movement, relative to stages 32 and 34, leading to a gradual peak in either the clockwise or anti-clockwise magnitude of movement, depending on the direction of the tail flick. Embryos at stages 32 and 34 had greater regularity in the frequency of tail flicking than embryos at stage 24 and these tail flicks were often too rapid for optic flow to track continuously in the image sequence resulting in sharp peaks in the negative and positive movements calculated. In stage 32 and 34 individuals optic flow also identified and tracked blood flow within the organism and this produced a low level of continuous negative and positive movements which were not present in the motion parameter data for stage 24 embryos in which blood flow was not visible. Stage 34 individuals had hatched from the egg capsule but motion analysis output did not show any difference between them and stage 32 embryos, as the organism displayed the same body flicking behavior. The main difference in motion parameters between stages 32 and 34 was caused by a more pronounced heart beat and blood flow in stage 34, producing more continuous low level negative and positive movements (Figure 2).

### *Radix balthica*

Embryos at all five developmental stages exposed to salinity treatments of 5, 10 or 15, with the exception of embryos at developmental stage E9 exposed to a salinity of 5, were statistically distinguishable from control embryos (Table 1; Figure 3). All developmental stages of *Radix balthica* used here exhibited a dose-dependent decreased frequency and magnitude of embryonic movements with increased salinity. Embryos from developmental stages E3, E4 and E6 exhibited fewer ciliary-driven oscillations than control embryos. Embryos at E3, which under control conditions undergo a “figure of eight” path within the egg, when exposed to salinity, rotated with little net movement within the egg. *Radix balthica* at stages E9 and E11 exposed to a salinity of 5 exhibited less gliding around the egg compared to control embryos, and this resulted in less positive and negative movement identified by optic flow as less change to the X and Y centre of mass motion parameters. When embryos at stages E9 and E11 were exposed to a salinity of either 10 or 15 they stopped gliding around the egg, using their cilia, resulting in only minor changes to X and Y centre of mass coordinates. However, despite the lack of overall movement in the egg, embryos in these treatments did continue to exhibit small scale movements of their muscular foot and shell which led to small peaks in the negative and positive movement parameters recorded by the motion analysis.

*Radix balthica* embryos under control conditions at the developmental stages; E3, E4, E6, E9 and E11 were all statistically distinguishable from each other with the exception of E9 from both E3 and E6 (Figure 3; Table 2). The movement patterns of *R. balthica* produced by optic flow were markedly different between early and late developmental stages due to the developmental transition from regular ciliary driven rotations to complex muscular movements more similar to those seen in adult snails (Figure 2). The manually observed rates of ciliary rotation in the three earliest stages (E3, E4 and E6) closely matched the cyclical patterns in negative and positive angles of rotation produced by optic flow.

The oscillatory pattern in clockwise and anti-clockwise movement became more variable as development progressed from ciliary rotation (E3, E4 and E6) to muscular movement (E9 and E11) producing marked differences in the frame-to-frame optic flow parameters. Optic flow was still able to accurately to track embryos exhibiting these more complex muscular movement behaviors, despite accurate manual quantification not being possible.

E3 stage embryos are spherical and exhibited a “figure of eight” type movement within the egg capsule, which produced a sinusoidal pattern in the X and Y coordinates of its centre of mass (Figure 2). E4 stage embryos are “crescent shaped” and occupy approximately three times the volume of the E4 stage, which resulted in the coordinates of the centre of mass having a frequency close to that of its rotational rate. There was some long term movement in the X and Y coordinates of the calculated centre of mass caused by lateral, as opposed to spinning, movement within the egg capsule. E6 stage embryos occupy approximately one third of the egg capsule and are significantly more asymmetrical than the E4 stage and also exhibit variable rates of spinning which leads to a less uniform pattern. In E9 stage embryos, cilia-driven rotational behaviour is largely absent and replaced by prolonged periods of apparent inactivity, during which the embryos undergo muscular contractions and flexing of the foot and shell. The loss of rotational movement is evidenced by the lack of a clear rhythm in the centre of mass time series replaced instead by a mostly linear pattern. Periods during which no large scale movements occur are interrupted by occasional gliding around the egg capsule. E11 stage embryos are much larger than E9 stage embryos, the former almost completely filling the egg capsule, however they exhibit similar patterns of behaviour to E9 embryos. This increase in size explains the decrease in the amount of “noise” surrounding the X and Y centre of mass from the E9 to E11 stage as the embryo has very little room to move within the egg capsule in the E11 stage which likely restricts small scale movement.

Manual quantification of embryonic behaviour (tail flick frequency for *Danio rerio* and *Xenopus laevis* embryos and spinning frequency for *Radix balthica*) was found to be less effective at distinguishing individuals exposed to stress treatments than the motion, and subsequent frequency, analysis presented here. Manual quantification of movement parameters could not distinguish treatments that were not distinguishable using this motion analysis technique. Furthermore, this technique was successfully able to distinguish some treatments that were not distinguishable using manual analysis (Table 1).

## Discussion

The application of motion analysis that we present here to detect the effects of abiotic stressors on different stages of embryos of three species incorporates data quantifying embryonic movements at a range of frequencies and so provides a holistic analysis of embryonic motion. This type of analytical approach may be applicable to a wide range of species, at various developmental stages, as it doesn’t rely on one particular type of movement, but rather being able to incorporate a diverse range of embryonic movements into a single analysis. This potential benefit is demonstrated here through the ability of this technique to distinguish environment-induced changes in motion in early molluscan developmental stages undergoing ciliary rotation, late molluscan development, during which the heart beat and slow muscular contractions are the prominent movement, and vertebrate developmental stages during which movement is mostly in the form of tail flicking or, in later development, the beating heart and associated blood flow. This technique was also better able to distinguish embryos exposed to stressors than analysis of manual quantification of movement patterns (Table 1).

While we have shown that this technique can be applied to embryos of two of the most common vertebrate model species and an invertebrate embryo, there is no reason why, in principle, such analysis cannot be extended to species belonging to other phyla (e.g. crustaceans and annelids). The spectral frequency analysis used here to analyse the frame-to-frame motion parameters, could be modified to target particular frequencies and refined for use on different species or to increase efficacy at detecting a particular behavioural response to a stressor. Refinement of the spectral analysis to target frequencies relevant for particular movements would enable this technique to be used as an automated toxicological assay able to quantify complex behavioral responses to stressors.

Image analysis and automated pattern recognition have been used to relatively good effect in reliable and quick species identification procedures [29-32] and the determination of developmental stages [33,34], but have not been used successfully to stage live animals in real time. The innovative use of motion analysis presented here was successfully able to distinguish many of the developmental stages studied and so demonstrates its potential application to an automated system for staging living, animal embryos. Although most biologists are probably accustomed to staging organisms prior to their use in an experiment the efficacy and reliability of developmental staging has long been questioned [35]. Automated image analysis is recognized as offering advantages over manual analysis; including greater consistency and accuracy and is less time consuming [32]. Therefore this approach could offer the benefits of automated image analysis for staging living embryos. The ability of this technique to distinguish stress induced changes and embryonic developmental stages also demonstrates its potential application to automate both, screening of short term stress responses and longer term effects on rates of development e.g. decreased developmental rate in long term exposure to a stressful environment.

## Conclusions

Image analysis is a key tool in biology for the extraction of biologically meaningful data from image datasets. Here we present a novel application of motion analysis coupled with spectral frequency analysis for providing a holistic measure of movement patterns. This technique is able to distinguish environmentally-induced alterations to movement patterns, and also individuals at different stages of development, and therefore could have applications in disciplines such as ecotoxicology, developmental biology and biotechnology.

## Methods

### Treatments

*Danio rerio* (obtained from a stock population at Plymouth University, UK) were cultured in glass beakers (vol. = 200 ml), containing aerated, deionized water at 27°C. Periodically water in the glass beakers was changed at which time dead embryos were removed. Embryos at 19, 21.5 and 33 h post fertilization (hpf) [15] were placed into 20 ml glass vials and exposed to either: 1.5% ethanol (1.5% EtOH (purity = 99.99%, Fisher, UK) in deionized water) for 30 minutes, a salinity of 5 (Instant Ocean® Salt in deionized water) for 60 minutes or to a control treatment of deionized water. Glass vials were sealed with microfilm to prevent losses by volatilization and maintained at 27°C. Embryos were transferred to 96 multiwell plates for image acquisition.

*Xenopus laevis* (obtained from Bristol University, UK) were cultured in glass beakers (vol. = 200 ml) containing aerated, deionized water at 23°C. Twenty minutes prior to image sequence acquisition embryos at stages 24, 32 and 34 [16] were transferred to 20 ml glass vials and exposed to either: a salinity of 20 or a control of deionized water and maintained at 23°C. Embryos were transferred to 96-multiwell plates prior to image acquisition.

*Radix balthica* embryos (obtained from a stock population at Plymouth University) at developmental stages E3, E4, E6, E9 and E11 were placed individually in multiwell plates (96 wells, volume = 0.3 ml/well) containing a control of Artificial Pond Water (APW) [36] or a salinity of 5, 10 or 15 (Instant Ocean® salt in APW). Embryos were exposed to treatments 10 minutes prior to image sequence acquisition.

### Imaging setup

Images from a 4 MP monochrome camera (Allied Vision Technology Pike 421B) operating with 1600 × 1200 pixels at 15 Hz for the *Danio rerio* and *Xenopus laevis* image sequence acquisition and with 1000 × 1000 pixels at 7.5 Hz for *Radix balthica*. The camera was connected to zooming optic lens systems (*Danio rerio* – Keyence VHZ100R; *Xenopus laevis* – Keyence VHZ20R; *Radix balthica* - Zoom 70 XL Optical System), which was inverted beneath a Prior Optiscan motorized XY stage, using a purpose-built frame, and controlled using Micromanager software package [37]. Ten minute long image sequences were recorded for six individuals from each of: *Danio rerio* at 19 h, 21.5 h and 33 h stages from a deionized water control, 1.5% ethanol and a salinity of 5 [15]; *Xenopus laevis* at stages 24, 32 and 34 in a control treatment of deionized water and a salinity of 5 [16]; *Radix balthica* at stages E3, E4, E6, E9, E11 in a control treatment of Artificial Pond Water [36] and salinities of 5, 10 and 15 [17] (Figure 3).

### Motion analysis

Off-line, the image sequence was analysed for optical flow using the OpenCV toolkit by applying the sparse optic flow algorithm [14]. This temporal tracking technique first extracts corner features from each image. It then attempts to register image features from frame to frame, and assigns a velocity variance for each. A feature with high variance is probably not being tracked accurately and may be rejected. All remaining features are candidates for measuring an embryo’s motion. To ensure optic flow was accurately tracking embryonic movement all image sequences were watched whilst the analysis was running.

Four measures of motion were extracted for each frame (by comparison with previous frames). These are positive and negative angles of rotation and rho-theta angular changes (in polar coordinates) to the centre of mass for the features tracked within the embryo (see Figure 1 for example). Note the yellow and red vectors are negative and positive rotations (feasible because the embryos are gelatinous and flexible) and the blue vector arrow is the frame to frame change to the centre of mass.

The method is not specific to the embryo and can be misled by the motion of other targets, such as the embryo’s egg capsule. However, this is normally infrequent. Frame-to-frame measurements from the motion analysis were saved to a CSV format file.

### Spectral frequency analysis

Frame-to-frame motion analysis data were analysed for spectral content using the Discrete Fourier Transform (DFT) [38]. This reveals cyclic frequency of motion information for each embryo. This is completed using the R language for statistical computing [27]. Data are collected over a 10 min period and so frequency analysis can reveal repetitive oscillations from 3.75 Hz to 10 min for *Radix balthica* (recorded at 7.5 fps) and 7.5 Hz to 10 min for *Danio rerio and Xenopus laevis* (recorded at 15 fps). These are calculated for each embryo image sequence. Specific wavelength bins were further analysed from the DFT. For *Danio rerio and Xenopus laevis* the following frequency bins were used:- [1]: 598 – 298 s; [2]: 200 – 149 s; [3]: 120 – 99 s; [4]: 85 – 54 s; [5]: 49 – 33 s; [6]: 29 – 20.5 s; [7]: 21 – 17 s; [8]: 16 – 15 s; [9]: 14 – 13 s; [10]: 12 – 11 s; [11]: 10 – 5 s; [12]: 4.9 – 3.5 s; [13]: 3.3 – 2.5 s; [14]: 2.4 – 1.7 s; [15]: 1.6 – 1 s; [16]: 0.9 – 0.33 s; [17]: 0.3 – 0.25 s; [18]: 0.24 – 0.1 s. Analysis of *Radix balthica*’s frame-to-frame motion analysis output was performed using 18 frequency bins for developmental stage comparisons - [1]: 598 – 299 s; [2]: 291 – 145 s; [3]: 120 – 100 s; [4]: 85 – 75 s; [5]: 66 – 60 s; [6]: 54 – 50 s; [7]: 46 – 43 s; [8]: 40 – 37 s; [9]: 35 – 30 s; [10]: 29 – 24 s; [11]: 20 – 17 s; [12]: 15 – 12.5 s; [13]: 12.2 – 10 s; [14]: 9.5 – 5 s; [15]: 2 – 1.1 s; [16]: 1 – 0.68 s; [17]: 0.66 – 0.5 s; [18]: 0.3 – 0.26 s, and 30 frequency bins for environmental stressor analysis - [1]: 300 – 150 s; [2]: 100 – 75 s; [3]: 60 – 50 s; [4]: 43 – 37.5 s; [5]: 33.5 – 30 s; [6]: 27.5 – 25 s; [7]: 23 – 21.5 s; [8]: 20 – 19 s; [9]: 17.5 – 16.5 s; [10]: 16 – 14.5 s; [11]: 13.5 – 12.5 s; [12]: 12 – 11 s; [13]: 10.5 – 9.5 s; [14]: 9 – 6.5 s; [15]: 6.3 – 5 s; [16]: 4.9 – 3.5 s; [17]: 3.3 – 2.5 s; [18]: 2.4 – 2 s; [19]: 1.9 – 1.5 s; [20]: 1.4 – 1 s; [21]: 0.9 – 0.8 s; [22]: 0.7 – 0.65 s; [23]: 0.6 – 0.5 s; [24]: 0.49 – 0.4 s; [25]: 0.39 – 0.34 s; [26]: 0.33 – 0.28 s; [27]: 0.27 – 0.22 s; [28]: 0.21 – 0.18 s; [29]: 0.17 – 0.15 s; [30]: 0.14 – 0.13 s. *Radix balthica* during its development performs movement types ranging from ciliary driven rotation to muscular driven crawling. As a result, to discern treatments more frequency bins were required for analysis of *Radix balthica* than for *Danio rerio or Xenopus laevis,* as movement differences fell within rather than between frequency bins*.* These extra frequency bins enhanced the frequency discrimination and therefore the detection of subtle changes to these complex movement behaviours.

The data resulting from the DFT comprises 72 parameters (18 frequency bins for each of positive and negative angles of rotation and rho-theta angular changes, in polar coordinates, to the centre of mass) for each individual. These data were transformed (Log X + 1) and for each species a Bray-Curtis similarity matrix was calculated. This matrix was used to generate Multidimensional Scaling (MDS) plots and ANOSIM [28] was used to test for the degree of similarity within groups of embryos from particular developmental stages (Primer-E).

## Competing interests

The authors have a patent pending, entitled: Method and system for determining characteristics of an embryo and uses thereof (UK Patent application number: GB 1016616.3; Date of filing 2/10/2010).

This work was funded by the Marine Institute at Plymouth University through the HEIF4 programme.

## Authors’ contributions

TB and OT carried out experimental work and bioimaging. PC carried out programming required for motion analysis and frequency analysis. TB, OT, SDR and JIS participated in the data analysis. All authors read and approved the final manuscript.
